# Aging and chronic kidney disease: epidemiology, therapy, management and the role of immunity

**DOI:** 10.1093/ckj/sfae235

**Published:** 2024-07-27

**Authors:** Yukun Tang, Jipin Jiang, Yuanyuan Zhao, Dunfeng Du

**Affiliations:** Institute of Organ Transplantation, Tongji Hospital, Tongji Medical College, Huazhong University of Science and Technology; Key Laboratory of Organ Transplantation, Ministry of Education; NHC Key Laboratory of Organ Transplantation; Key Laboratory of Organ Transplantation, Chinese Academy of Medical Sciences; Wuhan, China; Institute of Organ Transplantation, Tongji Hospital, Tongji Medical College, Huazhong University of Science and Technology; Key Laboratory of Organ Transplantation, Ministry of Education; NHC Key Laboratory of Organ Transplantation; Key Laboratory of Organ Transplantation, Chinese Academy of Medical Sciences; Wuhan, China; Institute of Organ Transplantation, Tongji Hospital, Tongji Medical College, Huazhong University of Science and Technology; Key Laboratory of Organ Transplantation, Ministry of Education; NHC Key Laboratory of Organ Transplantation; Key Laboratory of Organ Transplantation, Chinese Academy of Medical Sciences; Wuhan, China; Hubei Key Laboratory of Hepato-Pancreato-Biliary Diseases, Wuhan, China; Institute of Organ Transplantation, Tongji Hospital, Tongji Medical College, Huazhong University of Science and Technology; Key Laboratory of Organ Transplantation, Ministry of Education; NHC Key Laboratory of Organ Transplantation; Key Laboratory of Organ Transplantation, Chinese Academy of Medical Sciences; Wuhan, China

**Keywords:** aging, chronic kidney disease, immune system, management, therapy

## Abstract

Chronic kidney disease (CKD) is now an unquestionable progressive condition that affects more than 10% of the general population worldwide, and has emerged as one of the most important causes of global mortality. It is clear that the prevalence of CKD among the aging population is significantly elevated. It involves a broad range of complex and poorly understood concerns in older adults such as frailty, malnutrition, sarcopenia, and even cognitive and mental dysfunction. In kidneys, renal function such as glomerular filtration, urine concentration and dilution, and homeostasis of sodium and potassium, can be influenced by the aging process. In addition, it is worth noting that CKD and end-stage kidney disease patients often have accompanying activation of immune system and inflammation, involving both the innate and adaptive immune system. Based on this background, in this review article we attempt to summarize the epidemiological characteristics of CKD in the aging population, discuss the immunological mechanisms in aging-related CKD, and furnish the reader with processes for the therapy and management of elderly patients with CKD.

## INTRODUCTION

Chronic kidney disease (CKD) is now an unquestionable progressive condition that affects more than 10% of the general population worldwide, with a total number of >800 million patients [1]. The data from Global Burden of Disease studies shows that CKD has emerged as one of the most important causes of global mortality [2, 3]. Progressive CKD increases the risk of end-stage of renal disease (ESRD), cardiovascular disease and other lethal complications [4]. To date, there is no effective treatment for CKD until the patients reach a stage requiring renal replacement therapy. It is clear that the prevalence of CKD among the aging population is significantly elevated. In the last century, the improvement of healthcare has led to an increase in the population's age and an extension of life expectancy. The steady increase in the global elderly population poses a significant challenge for healthcare providers in dealing with age-related diseases [5]. Aging is a complex biological process characterized by a gradual decline in physiological functions and an increased susceptibility to diseases [6]. For the kidneys, aging not only leads to functional and structural changes but also significantly increases the risk of age-related kidney diseases due to the decline of the immune system with age. In the past 30 years in the USA, the number of patients over 75 years old with ESRD has tripled, increasing from 7.6% to over 20% [7]. Concurrently, due to the aging population and the rising prevalence of age-related diseases such as hypertension and diabetes, the elderly constitute the fastest growing segment of patients diagnosed with ESRD, and millions of them die due to the lack of kidney replacement therapy (KRT) [8]. ESRD involves a broad range of complex and poorly understood concerns in older adults such as frailty [9], malnutrition [10], sarcopenia [11], and even cognitive and mental dysfunction [12, 13]. Fundamentally, aging is due to cellular degeneration and faulty repair. In kidneys, renal function such as glomerular filtration, urine concentration and dilution, and homeostasis of sodium and potassium, can be influenced by the aging process [14].

It is worth noting that CKD and ESRD patients often have accompanying activation of the immune system and inflammation. In addition, the immune system also actively intervenes in the systemic inflammation present in CKD [15, 16]. In fact, elder adults with chronic organ disorders tend to develop a condition called “inflammaging,” which further increases the susceptibility to chronic disease progression [17], and which involves both the innate and adaptive immune system. For instance, death of tubular cells initiates an innate immune response. Damage-associated molecular patterns (DAMPs) which are released by necrotic cells activate identical pattern recognition receptors and further guide various types of inflammatory cells to aggravate the progress of cell deaths [18]. Aging of the adaptive immune system is mainly characterized by the loss of naïve T cells which results in a limited T-cell receptor (TCR) repertoire [19]. In other words, inflammaging is closely associated with CKD and also age-related comorbidities. While the data shows that in 2050, one in six people in the world will be aged ≥65 years. Therefore, the prevalence of CKD will also be elevated [20].

Based on this background, in this review article, we attempt to summarize the epidemiological characteristics of CKD in the aging population, discuss the immunological mechanisms in aging-related CKD, and furnish the reader with processes for therapy and management of elderly patients with CKD.

## EPIDEMIOLOGICAL CHARACTERISTICS OF CKD

### Definition

**Figure 1:**
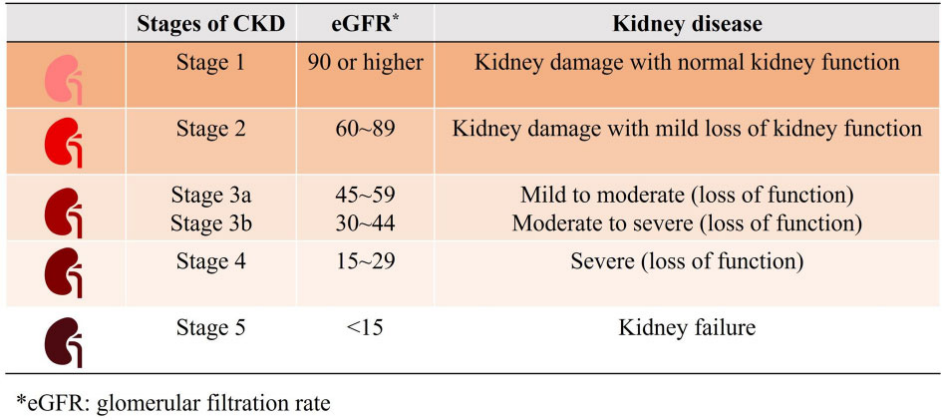
The definition of five stages of CKD.

According to the KDOQI (Kidney Disease Outcomes Quality Initiative) guidelines [21], CKD is defined by a reduced glomerular filtration rate (GFR) without regard to etiology, or by signs of kidney damage such as proteinuria, hematuria or abnormal imaging/biopsy findings. Usually, reduced GFR is defined as GFR <60 mL/min/1.73 m^2^ [22]. In the past two decades, the detection and monitoring of CKD improved, advocated by various professional organizations. Recently, most studies have used estimated GFR (eGFR, typically estimated from a filtration marker, such as serum creatinine or cystatin C) to determine the presence of CKD, whereas others have combined albuminuria, which is defined as an albumin-to-creatinine ratio of >30 mg/g and decreased eGFR [1]. Usually, a total of five stages of CKD were defined based on the eGFR (Fig. 1). In addition, to differentiate from acute kidney injury (AKI), CKD need include a “chronicity criterion” which is usually present for >90 days/3 months with specific implications for health [23]. Finally, standardized creatinine assays should be used to improve the accuracy of GFR estimates [24].

As previously mentioned, the conventional definition of CKD primarily relies on the identification of albuminuria or a decrease in the eGFR. For many years, the direct eGFR threshold of <60 mL/min/1.73 m^2^ has been widely adopted as the standard for defining CKD [25]. However, this standard does not take into account the natural aging process of the kidneys, and this oversight may affect the accurate diagnosis of kidney disease, particularly in extreme age groups, often leading to the overdiagnosis of kidney disease in the elderly and underdiagnosis in the young [26]. Based on these, some recent opinions support that it is necessary to adjust the diagnostic criteria for CKD for different age groups. They suggest that compared with the fixed threshold diagnostic criteria, age-adapted definition of CKD seems to more accurately reflect the physiological characteristics and renal health status of different age groups [27, 28]. Of cause, it is still an ongoing debate.

It is worth noting that the GFR progressively declines with age [29]. Statistically, the GFR in healthy adults after 60–65 years of age can decline to <60 mL/min/1.73/m^2^, depending on gender and attained age [30]. Furthermore, in the elderly and some other conditions, the estimating equations of eGFR are relatively inaccurate because of the impact of hypertension and cardiac function [31]. Previous studies confirmed that the prevalence of CKD was notably higher in elderly patients when assessed by plasma iohexol clearance than by creatinine-based eGFR equations [32, 33]. While the mechanism of the declining GFR with healthy aging are still not clearly understood. Chronically low protein intake which can lead to a physiologically reduced GFR may be one main reason [34]. Based on these, it is necessary for a more sensitive indicator or method to estimate kidney function in the aging population.

### Epidemiology and risk factors of CKD in elderly

CKD is a progressive disease that affects 10%–12% of the population. Although CKD prevalence depends on the method used to measure eGFR, which can be influenced by a normal physiologic decline with aging, it is well known that the prevalence of CKD is notably higher in elderly. In fact, almost half of adults aged 65–74 years old have five or more chronic health conditions, and this may reach 70% in individuals >85 years [35]. The data from several very large databases showed that the prevalence of CKD in elderly adults aged >65 years was approximately 44% [36, 37]. This is mainly because the structure and function of the kidney are significantly affected by the aging process. Once beyond age 50 years, the adult kidney presents a steepest decline in renal mass [14]. Nitta *et al*. summarized the morphological and microscopic changes in aging kidney mainly including: (i) fat and fibrosis scarring replacing the normal tissues; (ii) destructive and diffuse glomerular sclerosis; (iii) an increased proportion of globally sclerotic glomeruli; and (iv) increased arteriosclerosis, medial hypertrophy and arteriolar hyalinosis [38].

**Figure 2:**
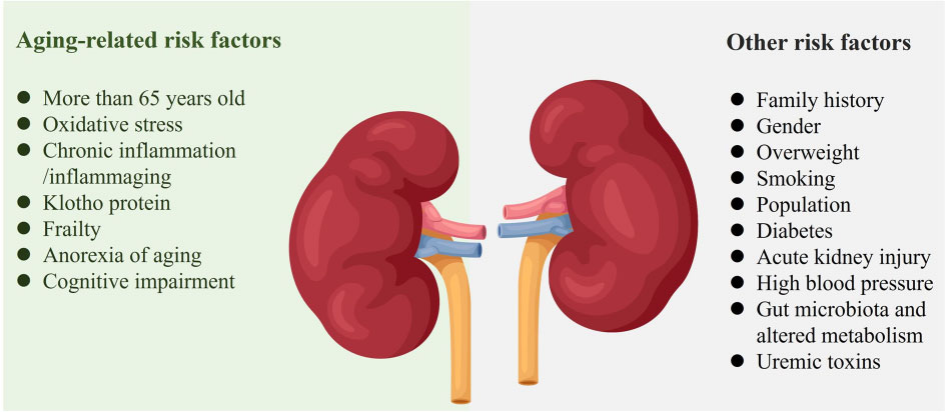
The aging-related and other risk factors of CKD.

Many genetic and environmental factors can increase the risk of CKD including the Klotho protein, gender, age, inflammation, altered metabolism, oxidative stress, microvascular damage, unhealthy living habit such as smoking, uremic toxins and kidney injury [39–42]. Notably, many risk factors are associated with aging (Fig. 2). For instance, the Klotho protein regulates many aging-related pathways, and the Klotho-deficient mice present the clinical phenotype of CKD, which also suggests that CKD is part of the diseasome of aging [43, 44]. Oxidative stress also plays a key role in the progression of CKD [45]. It can induce glomerular damage and renal ischemia. In addition, oxidative stress can lead to worsening frailty in elderly patients with CKD [46]. Furthermore, chronic inflammation is a vital risk factor of CKD, while elderly adults usually have chronic inflammation. Specially, some elements in the aging population such as frailty, anorexia of aging, and cognitive impairment are also significantly associated with CKD [47]. Worldwide, in patients with hemodialysis, the prevalence of frailty/pre-frailty varies from 43%–60% to 78% [48]. Cognitive impairment increases the risk of falls, worsening kidney function and mortality of CKD because of nonadherence to medications [49, 50]. These elements lead to the continued increasing number of patients with CKD in the aging population.

### Outcomes of CKD in elderly

**Figure 3:**
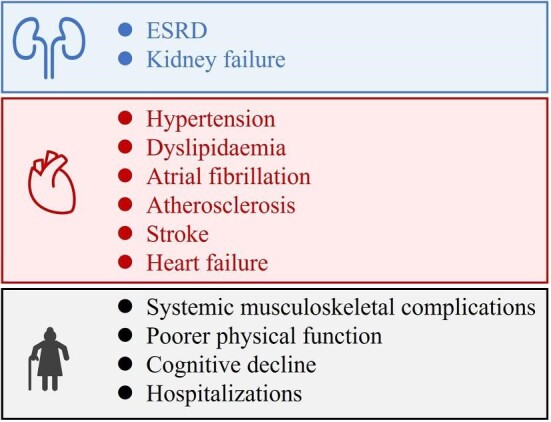
The clinical outcomes of CKD.

Substantial evidence has suggested that aging individuals with CKD face a high risk of severe outcomes including geriatric conditions such as functional impairment and cognitive decline, hospitalizations, ESRD and cardiovascular events, and even death (Fig. 3).

First of all, although CKD itself is a heterogeneous condition encompassing many diseases, it is consistently associated with cardiovascular disease risk and burden [51]. Patients with CKD usually have a higher prevalence of hypertension, dyslipidemia, atrial fibrillation, atherosclerosis, stroke and so on. A growing body of studies have demonstrated the disproportionate cardiovascular diseases risk incurred in patients with CKD. eGFR and albuminuria are the important biomarkers for increased risk of cardiovascular diseases [52, 53]. In addition, altered metabolism of solutes that accumulate in CKD patients may increase the risk of atherosclerosis [54]. Iron deficiency and anemia are the most common comorbidities in patients with CKD, which are associated with increased cardiovascular diseases–related mortality [55]. In the Action to Control Cardiovascular Risk in Diabetes (ACCORD) trial, in patients with diabetes and mild-to-moderate CKD, the risk of all-cause mortality was 97% higher, and the risk for cardiovascular mortality was 119% higher than in patients without CKD [56]. On the other hand, the risk of progression to ESRD was increased obviously in patients with CKD. However, a meta-analysis of 11 trials found that intensive blood pressure lowering in people with CKD could reduce the risk of progression to ESRD by 21% [57].

In the older population, patients with CKD are susceptible to systemic musculoskeletal complications, such as sarcopenia and CKD–mineral bone disease [58] because CKD affects bone metabolism through activating vitamin D deficiency, secondary hyperparathyroidism and abnormalities in bone-derived hormones [59]. Therefore, the National Kidney Foundation KDOQI guideline recommends that oral activated vitamin D treatments are given to people with CKD stage 3 and 4. Based on these, kidney transplantation (KT) may be a good choice for patients with CKD. A systematic review of 110 cohort studies found that compared with CKD patients with dialysis, kidney recipients had reduced mortality, cardiovascular events and better reported quality of life [60].

## IMMUNITY IN AGING-RELATED CKD

It is clear that the incidence rate of CKD is higher in older individuals. As a life-associated disease, CKD has shared features with other chronic diseases that increase in prevalence with age, including macromolecular damage, low-grade persistent inflammation, cellular senescence and stem cell dysfunction [61]. This chronic inflammatory response which has been named “inflammaging” or “immunosenescence.” Numerous studies found that many risk factors and mechanisms can increase the morbidity and mortality in older patients with CKD and ESRD. However, the mechanism of immunosenescence is still under investigation. Usually, individuals over 50 years old can present immunosenescence, which refers to the immune system changes including diminished immune response and chronic inflammation [62]; it has closely interrelation between the immune system and kidney function (Table 1).

**Table 1:** The interrelation between the immune system and kidney function.

Immune system	Class	Effector molecules
Innate immunity	TLRs	TLRs: TLR1–5 and 9, MyD88, IRF, IFN-β, TRIF, MAPKs, NF-κB
	NLRs	NLRP3, IL-1β, IL-18, NOD2, NLRC5
	RIG-I-like receptors	RIG-I, c-Myc, NF-κB
	CTLRs	CTLRs
Adaptive immunity	Naïve T cells, regular T cells, naïve and memory B cells, memory T cells, Th lymphocytes	TNF-α, IFN-γ, and IL-12, IL-4, IL-5

### Immunosenescence

Aging is a complex physiological phenomenon that leads to a variety of changes in multiple systems of the body [63, 64]. The most significant of these changes occur within the immune system, where both intrinsic and extrinsic factors shape the immune response, which undergoes profound alterations with age [65, 66]. In other words, immune responses are age-dependent, with cellular phenotypes and the composition of the immune system changing as a person ages. This is referred to as “immunosenescence,” which exhibits two contrasting characteristics: increased susceptibility to new infections and persistent low-grade systemic inflammation [67]. Consequently, patients with immunosenescence are at a higher risk of developing numerous degenerative diseases, particularly neurodegenerative disorders, cancer, cardiovascular diseases and autoimmune diseases [68–70]. Studies have shown that maintaining immune function can reduce the incidence of diseases and extend life expectancy [71]. Immunosenescence encompasses changes in both the innate and adaptive immune systems.

### Innate immune systems

#### Innate immune response cells

The innate immune system is composed of a variety of cells, including neutrophils, monocytes/macrophages, dendritic cells and natural killer (NK) cells [65, 66].

#### Neutrophils

Neutrophils make up 50%–70% of human white blood cells and play a crucial role in the innate immune system. They are the first cells to arrive at the site of an attack [72, 73]. With age, their numbers in the peripheral blood and bone marrow of the elderly remain stable, but their phagocytic and killing activities are reduced, and they are more susceptible to apoptosis [74].

#### Monocytes and macrophages

Monocytes express a wide range of pattern recognition receptors and play a key role in innate immune responses. Monocytes are activated upon pathogen stimulation through Toll-like receptors (TLRs), subsequently secreting pro-inflammatory cytokines and presenting antigens, thus exerting various immune effector functions [75]. Macrophages are tissue-resident cells that can phagocytose pathogens and initiate inflammatory signaling cascades. Monocytes and macrophages are considered core components in promoting low-grade chronic inflammation during immunosenescence [76]. Macrophages are thought to have two distinct phenotypes, classical (M1) and alternative (M2), based on their different inflammatory responses and cytokine release, including tumor necrosis factor (TNF)-α, interleukin (IL)-1β and IL-12 [65]. The monocyte/macrophage lineage also shows age-related changes, with most effector functions of these cells decreasing with age, including cytotoxicity, intracellular killing and antigen presentation [77, 78]. Recent studies have found an increase in the inflammatory CD14^+^CD16^+^ monocyte subset in the elderly, leading to increased production of pro-inflammatory cytokines in a resting state and reduced production upon stimulation [79]. This is because, although the monocyte population is larger in the elderly, their TLR expression and biological response are impaired, and when stimulated through TLRs, they produce fewer pro-inflammatory cytokines [such as IL-1β and interferon (IFN)-γ] compared with younger cells [80]. Meanwhile, M2 monocytes, marked as anti-inflammatory monocytes, show a significant age-related decrease in healthy elderly individuals [81].

#### Dendritic cells

Dendritic cells (DCs) are the most effective antigen-presenting cells with surface TLRs. Upon antigen stimulation, DCs capture, process and present pathogen antigens through major histocompatibility complex II and release cytokines, thereby activating naïve T cells and initiating adaptive immunity [82]. Several studies have shown age-related impairment in DC antigen presentation and T-cell activation in the elderly, characterized by reduced antigen presentation function, impaired endocytosis and decreased production of chemokines [83–85].

#### NK cells

NK cells are key cells in the innate immune system for identifying and eliminating virus-infected and abnormal cells, including tumor cells. Based on the expression levels of surface molecules CD56 and CD16, NK cells can be divided into two subgroups: CD56^bright^ cells, an immature subgroup with high proliferative activity and the ability to produce a range of cytokines, such as IFN-γ, TNF-β and IL-10, as well as chemokines like RANTES and macrophage inflammatory protein-1α. In contrast, CD56^dim^ cells are a mature subgroup with higher cytotoxic activity and lower cytokine production capabilities [86]. With aging, the CD56^bright^ subgroup decreases and the CD56^dim^ subgroup increases, which may be due to the degeneration of hematopoietic stem cells in the bone marrow. Although the overall cytotoxicity of NK cells does not significantly decrease in healthy elderly individuals, the activity of each NK cell is reduced compared with younger individuals [65, 87, 88].

#### Effector molecules of innate immune systems

The innate immunity is characterized by non-specific recognition of self and non-self stimuli through pattern recognition receptors (PRRs) [62]. PRRs are soluble cell-associated receptors specialized in virus, bacteria and fungi recognition of pathogen-associated molecular patterns (PAMPs) and danger-associated molecular patterns (DAMPs). PAMPs and DAMPs activate the inflammatory response through the synthesis of proinflammatory cytokines via nuclear factor (NF)-κB and activator protein-1 (AP1) [89]. Once PRRs are activated, they further activate immune cells, the complement system and the coagulation system [90]. Innate immunity plays an important role in many diseases including CKD. For instance, NLRP3 inflammasome, the most widely studied factor in innate immunity, has been demonstrated to be associated with CKD following multiple pathways [91, 92]. In addition, several classes of PRRs including TLRs, C-type lectin receptors, nucleotide-binding oligomerization domain (NOD)-like receptors (NLRs), retinoic acid-inducible gene-I-like receptors (RIG-I-like receptors) also play key roles in CKD and immunosenescence.

#### Toll-like receptors

Recently, a total of 11 members of TLRs have been found in humans including TLR1–11, and 13 members in mouse including TLR1–13. Among them, TLR2 and TLR4 are the most widely reported. They are usually promoted by the cytoplasm protein Myeloid differentiation primary response protein 88 (MyD88) and further activate NF-κB and the Interferon regulatory factor family (IRF) [90]. Much evidence has demonstrated that TLRs are key factors in aging-related CKD. For instance, it has been demonstrated that activation of TLR3/IFN-β pathway significantly affects the progression of CKD through regulating the expression of RIG-I and melanoma differentiation associated gene 5 (MDA5) protein by a deubiquitinase Cylindromatosis (CYLD) [93]. In addition, in aging cells, the stimulation of TLR3 and TLR4 can induce TIR-domain-containing adapter inducing interferon-β (TRIF)-mediated necroptosis, which is a process regulating cell necrosis. Necroptosis causes to a massive DAMP release and induce relevant tissue inflammation [94]. In fact, TLR2 and TLR4 play a vital role in CKD and other infection-associated renal diseases. Many studies have showed that TLR2 and TLR4 are the most common TLRs activated in CKD [95, 96]. In AKI mice models, lipopolysaccharide can bind to TLR4 and induce proximal tubular paracellular leakage of the glomerular filtrate [97]. TLR4 expression in CKD was positively correlated with IL-6 and monocyte chemoattractant peptide protein 1 (MCP-1) and enhanced the downstream MAPKs, NF-κB and TNF-α expression during the progressive loss of renal function. For TLR2, murine models show that TLR2 is a key mediator of senescence by promoting cell cycle arrest and regulating the induction of acute serum amyloids A1-2 that can function as DAMPs [98]. TLR9 plays as a proinflammatory receptor that mediates the AKI-to-CKD transition through regulated macrophages [99]. A genetic study provides evidence that in the Han Chinese population, those who carry the C and A alleles at SNPs T-1237C and G1635A in the *TLR-9* gene appear to be more susceptible to the development of CKD [100]. Furthermore, in aging rat models, TLR1–5 have been found associated with NF-κB signaling activation [101]. Overall, this evidence suggests that TLRs are important in both renal chronic diseases and renal aging.

#### NOD-like receptors

NOD-like receptors (NLRs) are cytoplasmic soluble proteins acting as PAMP receptors. Among them, NLRP3 is the most widely studied. NLRP3 is also known as the inflammasome and is an intracellular NLR complex. NLRP3 has been demonstrated associated with pyroptosis by the release of cytoplasmic IL-1β and IL-18, triggering necroinflammation [102]. NLRP3 deficiency results in decreased systemic inflammation and reduced immune cell activation. As we know, inflammaging is associated with the accumulation of damage-related molecules. The activation of NLRP3 is induced by many endogenous triggers including damaged mitochondria and reactive oxygen species, and aggregated proteins in the process of inflammaging [103]. A recent genetic study provided evidence about the association between *NLRP3* rs10754558 and susceptibility to CKD [104]. Therefore, some small molecule inhibitors targeting the NLRP3 inflammasome are potential agents for the treatment of CKD, such as glibenclamide, under clinical application, which has been demonstrated to inhibit CKD and renal fibrosis [105–107]. In addition, the expression of NOD-containing protein 2 (NOD2), NOD-like receptor family CARD domain containing 5 (NLRC5) and NLRP3 was significantly higher in patients with renal injury and anti-neutrophil cytoplasmic antibody–associated vasculitis [108], which also affect the progress of CKD. As a whole, TLRs and NLRs are activated in CKD via a member of inflammatory pathways, along with a series of immune cells.

#### RIG-I-like receptors

RIG I-like receptors are cytoplasmic sensors which are activated by viral dsRNA via the production of type I IFN and the activation of DCs. A recent study observed that endogenous retroviruses could active the cytosolic nucleotide sensors such as RIG-I in kidney tubule cells and induce the renal fibroinflammation [109]. Similar studies also showed that RIG-I aggravated renal interstitial fibrosis and CKD via c-Myc-mediated fibroblast activation [110]. Furthermore, in 12-month-old senescence-accelerated mouse models, the downregulation of Klotho and induction of the RIG-I/NF-κB signaling pathway were observed associated with aging-related inflammation and the development of early-stage CKD [111], suggesting that RIG-I plays a key role in renal inflammaging. However, more evidence need to be explored to demonstrate the correlation between RIG-I-like receptors and aging-related CKD.

#### C-type lectin receptors

C-type lectin receptors (CTLRs) are a class of antigen-uptake receptors mainly expressed by myeloid cells via their carbohydrate-recognition domain for internalization and subsequent presentation to T cells. Recent studies have demonstrated that CTLR dysregulation could lead to the development of autoimmune diseases, virus infection, allergy and cancers [112, 113]. Although no studies have verified the correlation between CTLRs and aging-related CKD, some research showed that CTLRs were associated with inflammaging and renal vasculitis [90].

#### Adaptive immunity

Unlike the innate immune system, the adaptive immune system generates immunological memory following an initial response to a specific pathogen [62]. The adaptive immune system can lead to an enhanced and rapid response when re-encountering that pathogen. However, in patients with declining kidney function, this system may be characterized by a reduction in the output of naïve T/B cells, an abnormal phenotype in mature cells and an accumulation of memory cells [114]. It is worth noting that patients with CKD and the elderly exhibit similar phenomena in the adaptive immune system, such as premature thymic involution, which leads to a significant decrease in the production of naïve T cells [115, 116]. Although decrease can be partially compensated by peripheral homeostatic proliferation, as age increases, T cells are repeatedly triggered by the same antigens throughout a lifetime of chronic antigen exposure. This leads to a continuous reduction in the naïve T-cell pool, ultimately resulting in a loss of diversity in the TCR repertoire, which causes an increased susceptibility to new infections, a diminished response to vaccinations and a poorer memory for previously encountered pathogens in the elderly [117, 118]. In addition, studies have demonstrated that the number of cells in the CD4 and CD8 compartments and T-cell proliferation decline in the progression from mild renal dysfunction to ERSD [119, 120]. On the other hand, naïve and memory T cells are active in patients with ERSD, and show increased susceptibility to apoptosis [121]. In addition, T-helper (Th) lymphocytes also play a key role in controlling the immune response through producing various cytokines including TNF-α, IFN-γ, and IL-12 (produced by Th1 cells) and IL-4 and IL-5 (produced by Th2 cells) [122]. The naïve B cells develop to memory B cells once they have been stimulated by antigens, and further differentiate to plasma cells with large amounts of a specific immunoglobulin. All the variations are compatible with the concept of premature immunological ageing.

#### Immunosenescence and CKD

**Figure 4:**
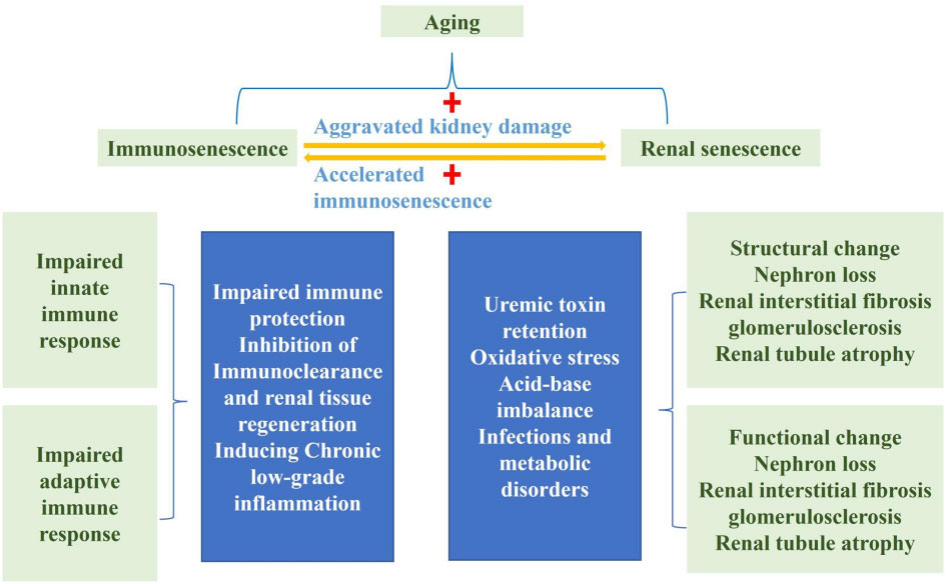
An interplay between immunosenescence and the development and progression of CKD.

Immunosenescence is the functional decline of the innate and adaptive immune systems associated with aging. In the state of inflammaging, age-related impairments in immune function can lead to compromised immune protection and persistent low-grade chronic inflammation. This reduces the elderly's effective response to new infections, cancer and endogenous tissue damage, thereby increasing their susceptibility to infections, cancer, cardiovascular diseases and autoimmune diseases [67, 123]. Studies have shown that a long-term, sterile, low-grade inflammatory state is almost universally present in the elderly. While acute inflammation is considered a protective process against harmful pathogens, persistent unresolved inflammation may be detrimental [123, 124]. The most common chronic diseases associated with aging, such as atherosclerosis, obesity, diabetes and neurodegenerative diseases, are all related to a process of low-grade continuous inflammation. Inflammatory aging has also been confirmed as the most significant risk factor for these chronic diseases [125, 126]. Among them, CKD and ESRD are significant chronic diseases posing a severe threat to human survival in the 21st century. According to statistics, more than 1.2 million people died from CKD in 2017, indicating a substantial increase in global age-adjusted prevalence and mortality rates over the past 20 years. The Global Burden of Disease Study reported a 29.3% increase in global CKD prevalence from 1990 to 2017 (range 26.4%–32.6%) [7, 127]. Studies have shown that immunosenescence appears to be more pronounced in patients with kidney disease than in healthy controls, characterized by the accumulation of immunologically senescent cells (such as CD28^–^ T cells, CD14CD16 monocytes) and the production of pro-inflammatory cytokines [128, 129]. When senescent cells accumulate in the kidney and induce chronic low-grade inflammation, they exacerbate kidney damage, accelerate kidney aging and further increase the susceptibility of the elderly to kidney diseases. In addition, immunosenescence inhibits immune clearance and renal tissue regeneration, increasing the risk of permanent kidney damage, infections and cardiovascular events in patients with kidney disease, worsening prognosis and even affecting the effectiveness of renal replacement therapy [130]. CKD patients often exhibit cellular changes characterized by chronic inflammation, advanced cellular senescence and immune system dysfunction [128]. In the persistent uremic environment of CKD patients, the long-term retention of toxins can lead to chronic inflammation, and premature aging of the immune system may be induced and accelerated [131]. Furthermore, oxidative stress, acidosis, infections and metabolic disorders may accelerate immunosenescence in CKD patients, and subsequently, exacerbated immunosenescence can promote disease progression and increase susceptibility to infections. In summary, age-dependent changes in the immune system associated with aging play a crucial role in the pathogenesis of kidney disease. Therefore, exploring the underlying processes and mechanisms of age-related changes in the immune system and studying the interaction between immunosenescence and kidney disease may provide new avenues and insights for the development of new treatment methods for kidney diseases [114]. The interplay between immunosenescence and the development and progression of chronic kidney disease is detailed in Fig. 4.

## MANAGEMENT FOR ELDERLY PATIENTS WITH CKD

CKD is a progressive and irreversible condition. Once it advances to the stage of ESRD, interventions such as dialysis or KT become necessary. However, these treatments are often costly and not readily accessible [132]. Furthermore, the life expectancy for all age groups on dialysis was found to be shorter compared with that of the general population [133]. Over the years, for slowing or preventing the progression of CKD to ESRD and reducing mortality, we have established various management guidelines to help to optimize the management of patients with CKD. Although numerous management guidelines have been developed by different organizations and expert panels, current guidelines for the management of CKD universally emphasize the importance of early detection and intervention, as well as a multidisciplinary approach. (i) Early detection and intervention are recommended to slow or halt disease progression. The main measures include regular monitoring of kidney function, controlling blood pressure, blood sugar and lipid levels, as well as lifestyle changes such as a healthy diet, regular exercise and smoking cessation. Additionally, medication is advised to manage complications like anemia, bone disease and cardiovascular disease. (ii) The importance of a multidisciplinary approach to treating CKD is highlighted. It is suggested that nephrologists, primary care physicians, dietitians, pharmacists and other healthcar professionals collaborate in the diagnosis and treatment of patients with CKD to provide comprehensive and coordinated management [134, 135]. Relevant research indicates that with proper management and timely intervention, the progression of CKD can be slowed or halted, complications and symptoms can be controlled, the onset of ESRD can be prevented or delayed, and the quality of life and prognosis for patients can be effectively improved [136–138]. Furthermore, although KT has the best health outcome and is the most economical KRT for treating ESRD, the severe supply and demand imbalance caused by organ shortage means that most ESRD patients cannot obtain a transplant opportunity. From 1990 to 2017, the number of global KT recipients with ESRD increased by 34.4% [127]. With the significant increase in demand for KTs, the average age of people on the transplant waiting list is also increasing. Elderly CKD patients often have various chronic diseases and may lose their lives due to various complications during the waiting period. For elderly kidney disease patients, standardized conservative management during the waiting period is very necessary, which can extend the survival period and buy as much time as possible for KT [139]. The main management measures currently recommended are summarized in the Table 2 for reference.

**Table 2:** The main management measures currently recommended for elderly patients.

Risk factor management	Reducing risk of cardiovascular disease	(i) Control blood lipids: it is recommended that patients aged 50 years or older with CKD be treated with a low- to-moderate dose statin regardless of low-density lipoprotein cholesterol level. (ii) Control blood pressure: goal systolic and diastolic blood pressures of <140 mmHg and <90 mmHg, respectively, among adults with CKD based on expert opinion. The KDIGO guidelines further recommend that adults with urine ACR of at least 30 mg/24 h (or equivalent) have systolic and diastolic blood pressures maintained below 130 mmHg and 80 mmHg, respectively	
	Management of hypertension	In diabetic patients with urinary ACR of at least 30 mg/24 h or in any adult, ACE inhibitors or ARBs are recommended, given the associated risk of hyperkalemia and AKI. Dual treatment with ACE inhibitors and ARB is usually avoided. Aldosterone receptor antagonists may also be considered for patients with albuminuria, refractory hypertension or heart failure with reduced ejection fraction	
	Management of diabetes mellitus	Strict control of blood sugar levels can help prevent or delay the onset of kidney damage; most guidelines recommended a goal hemoglobin A1c of 7.0%	
Lifestyle management	Exercise regularly		
	Encourage smoking cessation		
	Diet management	A low-salt, low-phosphorus and low-protein diet is recommended to reduce renal load and control symptoms. For adult patients with stage G4–G5 CKD, protein intake should be reduced to <0.8 g/kg/day, and for other adults with CKD at risk of progression, protein intake should be reduced to <1.3 g/kg per day, with a lower dietary acid load (e.g. eating more fruits and vegetables, eating less than 1.3 g/kg per day). Eating less meat, eggs and cheese may also help prevent kidney damage. For patients with hypertension, proteinuria or fluid overload, a low-sodium diet (usually <2 g per day) is recommended	
Drug administration	Reduce the use of nephrotoxic drugs		
	Pay attention to drug dose adjustment, drug metabolism and drug interactions		
Regular renal function monitoring and complication management	Regular renal function monitoring	(i) Once CKD is diagnosed, it is recommended to monitor eGFR and albuminuria at least once a year; (ii) high-risk patients: monitor these measures at least twice a year; (iii) extremely high-risk patients should be monitored at least three times a year (laboratory screening includes measurement of complete blood count, basic metabolic panel, serum albumin, phosphate, parathyroid hormone, 25-hydroxyvitamin D and lipid panel)	
	Treatment complications	Anemia	Rule out other causes of anemia: iron deficiency, vitamin B12 deficiency, folate deficiency, occult bleeding
		Mineral and bone disorder	Consider phosphate-lowering therapy (e.g., calcium acetate, sevelamer, iron-based binders) and vitamin D supplementation
		Hyperkalemia	Low-potassium diet, correction of hyperglycemia and acidemia, consider potassium binders
		Metabolic acidosis	Oral bicarbonate supplementation (e.g., sodium bicarbonate, baking soda or sodium citrate/citric acid) for values persistently <22 mmol/L
Referral to a nephrologist and timing of KRT	KDIGO guidelines recommend that when eGFR falls below 30 mL/min/1.73 m^2^ (stage G4) and/or urine ACR increases above 300 mg/24 h (stage A3), patients with CKD should be referred to a nephrologist for timely dialysis and kidney transplant evaluation		
			

ACR, albumin to creatinine ratio; KRT, kidney replacement therapy.

## THERAPY FOR ELDERLY PATIENTS WITH CKD

### Drugs

Although several drugs and treatments have been discovered in the past 20 years to reduce the risk of CKD progression, no drug class has provided a reduced risk of mortality in patients with CKD or slowed CKD progression [140]. As we known, angiotensin receptor blockers (ARBs) and angiotensin-converting enzyme (ACE) inhibitors have been commended as the only agents for patients with CKD and hypertension [141]. Previous Reduction of Endpoints in NIDDM with the Angiotensin II Antagonist Losartan (RENAAL) and Irbesartan Diabetic Nephropathy Trial (IDNT) studies showed that ARBs could reduce the risk of the composite renal endpoint by 16% and 20%, respectively [142, 143]. In addition, a randomized controlled trial compared the efficacy and safety of Shenyankangfu Tablet (SYKFT), a Chinese patent medicine, in decreasing proteinuria and the slowing the progression of CKD. Compared with the standard drug losartan potassium, which was one of the earliest ARBs used to treat CKD and decrease proteinuria, SYKFT could decrease the proteinuria, with no change in the rate of decrease in the eGFR [144]. For ACE inhibitors, several clinical studies demonstrated that compared with placebo, ACE inhibitors could slow the progression of CKD [145, 146]. In addition, some recent randomized studies investigated that sodium–glucose cotransporter 2 (SGLT2) inhibitors could significantly reduce the risk of clinically relevant renal outcomes including loss of kidney function, eGFR decline, worsening of albuminuria, new ESRD, death from renal causes and/or a renal composite outcome in patients with type 2 diabetes (T2D). However, only 7%–26% of participants in the studies had an eGFR of <60 mL/min/1.73 m^2^, so they did not evaluate treatment benefits of SGLT2 inhibitors in patients with CKD only [147–149]. Subsequent studies showed that SGLT2 could significantly reduce the risk of CKD progression with diabetic kidney disease (DKD) as well as nondiabetic CKD with the anti-diabetic drug dapagliflozin [150–152]. However, as we know, there has been no trial to evaluate the effect of SGLT2 inhibitors in patients with CKD only. In addition, to date, no drug has provided a reduced risk of mortality in patients with CKD only.

### Dialysis

Once patients with CKD present kidney failure, patients can choose dialysis including hemodialysis or peritoneal dialysis. The clinical symptoms and signs of kidney failure usually include serositis, acid–base/electrolyte abnormalities and pruritus. In addition, typical uremic symptoms generally occur at a low GFR values ranging between 5 and 10 mL/min/1.73 m^2^ [153]. Between 1980 and 2012, older patients (aged 65–74 years) initiating dialysis increased by 47% while those aged ≥75 years increased by 300% [154, 155]. While older persons usually have several comorbidities and a physiologically decreased value of eGFR, so there sometimes might be a less tight link between the true disease and the clinical phenotypes. Furthermore, it is worth noting that elderly patients have a higher early mortality rate within 3 months of initiating dialysis than younger patients [156]. This may be because dialysis can worsen cognitive function, which further worsens the quality of life, and increases hospitalizations and poor healthcare outcomes. A large-scale meta-analysis including 13 065 Korean patients (age ≥65 years) with hemodialysis or peritoneal dialysis showed that a higher risk for death in elderly patients receiving peritoneal dialysis than in those receiving hemodialysis [157]. Though some studies support that peritoneal dialysis has some quality-of-life advantages as it is a home-based treatment, such as fewer hospital visits, not requiring vascular access, avoiding hemodynamic instability and preserving residual function for a long time [158], older dialysis patients are less likely to be treated with peritoneal dialysis. Gadelha *et al*. performed a randomized controlled trial to investigate whether pre-dialysis resistance training (RT) could improve complications including sarcopenia and inflammatory profile in older patients with CKD [159]. The study found that 24-week RT could significantly improve the sarcopenia status, inflammatory profile and anemia biomarkers. Even so, older dialysis patients differ from younger patients, typically initiating dialysis at lower body mass index and higher eGFR levels, and having more comorbid conditions, and higher admission and mortality rates [160].

### Kidney transplantation

For most patients with CKD, KT is the best choice for treatment once the disease progresses to ESRD. KT improves quality of life and life expectancy, and reduces the financial burden in comparison with dialysis [161]. Due to the heightened risk of age-related kidney deterioration and an increased likelihood of comorbidities, the elderly population represents the fastest growing demographic of KT recipients (KTRs) [162]. In fact, recipient age can be an appropriate consideration, and transplant coordinators can allocate “marginal donors” to elderly recipients [35]. Rao *et al*. observed the outcomes among 5667 individuals aged ≥70 years old over an 18-month period and found that compared with waitlisted patients who received dialysis, the relative risk of death was 56% lower for transplant patients (*P *< .01) [163]. Studies from Australia, the UK and Asia showed similar results [164–166]. Therefore, older patients with ESRD can derive survival and quality-of-life benefits from KT. However, older recipients with KT have a higher risk of cancer, infection and cardiovascular diseases [167]. In addition, these patients have lower rejection rates, more medication side effects and higher mortality [162]. Yoo *et al*. performed a multicenter cohort study included 3565 KTRs in Korea, and observed that although the death-censored allograft survival rate did not differ between elderly and younger KTRs, the overall survival rate of the elderly KTRs was significantly lower than that in younger group [168]. A recent study from Chadban *et al*. also observed that, compared with remaining on dialysis, elderly KTRs incurred an increased risk of early post-transplant mortality [169]. Over a median follow-up of 1.7 years, mortality was 38% lower for KTRs (95% confidence interval 0.41–0.94, *P *= .02) compared with patients with dialysis. KTRs may had progressively superior survival rates after 5 years. However, a latest meta-analysis form Artiles *et al*. appraised the outcomes of KT in patients >70 years old. The data showed that the rate of delayed graft function, acute rejection, death with functioning graft and graft loss were not inferior in elderly recipients [170]. In addition, Hammad *et al*. analyzed the patient demographic data, and the 1-, 5- and 10-year patient and graft survival rates of patients aged ≥65 years who received KT from living or deceased donors, and found that the outcomes were better in patients who received kidneys from living donors [171]. Therefore, more evidence needs to be collected to evaluate the long-term prognosis in KTRs in elderly population. Overall, although a KT has its advantages in terms of survival, costs and quality of life, existing data show that the life expectancy of transplant patients is one-third shorter compared with the life expectancy of the general population [133]. Based on this, we can say that KT is far from an ideal solution for CKD, and prevention of CKD is key.

## CONCLUSIONS

Recent studies have revealed that CKD has become a leading public health problem. It is well known that the prevalence of CKD is notably higher in the elderly. Aging individuals with CKD face a higher risk for severe outcomes such as ESRD, cardiovascular diseases, geriatric conditions and even death. Macromolecular damage, low-grade persistent inflammation, cellular senescence and stem cell dysfunction in the kidney and the vascular system all concur in determining the unique severity of inflammation in CKD and increase in prevalence with age. Chronic inflammatory response is activated including innate and adaptive immunity and plays a key role in CKD and immunosenescence. Patients with CKD can accept the treatment of drugs, dialysis and KT. In the elder population, compared with CKD patients with dialysis, kidney recipients had reduced mortality and cardiovascular events, and better reported quality of life.

## Data Availability

No new data were generated or analyzed in support of this research.
